# Near fatal posterior reversible encephalopathy syndrome complicating chronic liver failure and treated by induced hypothermia and dialysis: a case report

**DOI:** 10.1186/1752-1947-3-6623

**Published:** 2009-03-26

**Authors:** Rashmi Chawla, Daniel Smith, Paul E Marik

**Affiliations:** 1 Division of Pulmonary and Critical Care Medicine, Thomas Jefferson University, Philadelphia, PA, USA; 2 Medical Intensive Care Unit, Thomas Jefferson University Hospital, Philadelphia, PA, USA

## Abstract

**Introduction:**

Posterior reversible encephalopathy syndrome is a clinico-neuroradiological entity characterized by headache, vomiting, altered mental status, blurred vision and seizures with neuroimaging studies demonstrating white-gray matter edema involving predominantly the posterior region of the brain.

**Case presentation:**

We report a 47-year-old Caucasian man with liver cirrhosis who developed posterior reversible encephalopathy syndrome following an upper gastrointestinal hemorrhage and who was managed with induced hypothermia for control of intracranial hypertension and continuous veno-venous hemodiafiltration for severe hyperammonemia.

**Conclusion:**

We believe this is the first documented case report of posterior reversible encephalopathy syndrome associated with cirrhosis as well as the first report of the use of induced hypothermia and continuous veno-venous hemodiafiltration in this setting.

## Introduction

Posterior reversible encephalopathy syndrome (PRES), first described by Hinchey and colleagues in 1996, is a clinico-neuroradiological entity characterized by headache, vomiting, altered mental status, blurred vision and seizures with neuroimaging studies demonstrating white-gray matter edema involving predominantly the posterior region of the brain [1]. PRES is most commonly associated with hypertensive encephalopathy, eclampsia, porphyria and immunosuppressive or cytotoxic drugs [1,2]. Magnetic resonance imaging (MRI) is the diagnostic test of choice, demonstrating hyperintense echoes on T2-weighted images of the parietal-occipital lobes; however, the cerebellar hemispheres, basal ganglia, frontal lobes and brainstem are often involved. While usually completely reversible, a delay in the diagnosis and treatment may result in death or irreversible neurological sequela [1,3]. We present a patient with liver cirrhosis who developed PRES following an upper gastrointestinal hemorrhage and who was managed with induced hypothermia for control of intracranial hypertension and continuous veno-venous hemodiafiltration (CVVHDF) for severe hyperammonemia.

## Case presentation

A 47-year-old Caucasian man with a past medical history significant for noninsulin-dependent diabetes mellitus, hepatitis C, past alcohol abuse and cirrhosis was admitted to our medical intensive care unit with an upper gastrointestinal bleed. On presentation, his blood pressure was 104/70mmHg, heart rate 137 beats per minute, temperature 37.2°C and respiratory rate 24 breaths per minute. On examination, he was pale and icteric, and had a mildly distended abdomen with no discernable organomegaly. Cardio-respiratory examination was normal. He was confused and agitated with no focal neurological signs. His white blood count was elevated at 23.7 × 10^9/^/L with 24% bands, with a hemoglobin of 78g/L and platelets of 83 × 10^9^/L. Albumin was 19g/dl (normal 32 to 49g/L), total bilirubin 83.3μmol/L (normal 3.4 to 20.4μmol/L), aspartate aminotransferase (AST) 32 IU/L (normal 7 to 42 IU/L), alanine aminotransferase (ALT) 35 IU/L (normal 1 to 45 IU/L), alkaline phosphatase (ALP) 32 IU/L (normal 25 to 120 IU/L), international normalized ratio (INR) of 1.84, arterial ammonia 151μmol/L (normal 11 to 35μmol/L) and lactate of 6.1mmol/L (normal 0.6 to 1.7mmol/L). His blood urea nitrogen (BUN) and creatinine were 9mmol/L and 124μmol/L, respectively.

**Figure 1:**
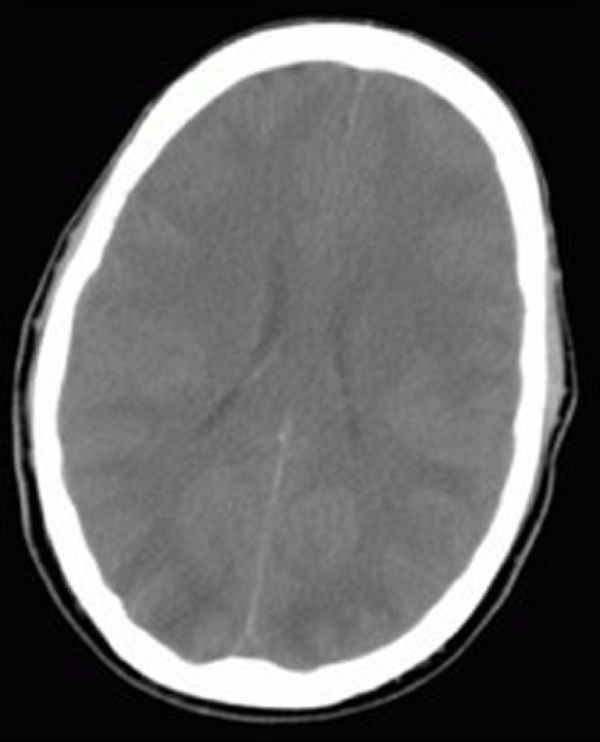
Non-contrast computed tomography showing diffuse cerebral edema.

**Figure 2:**
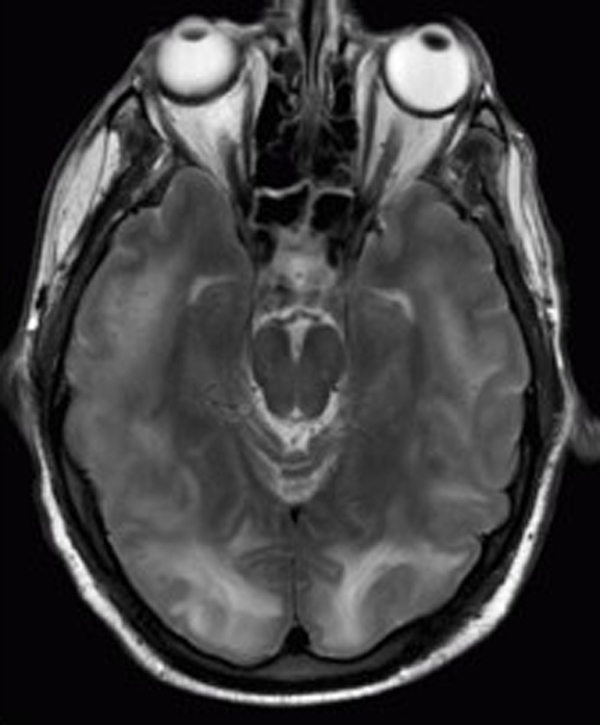
Magnetic resonance image showing multiple cortico-subcortical areas of hyperintense signal involving the occipital and parietal lobes bilaterally and pons

**Figure 3:**
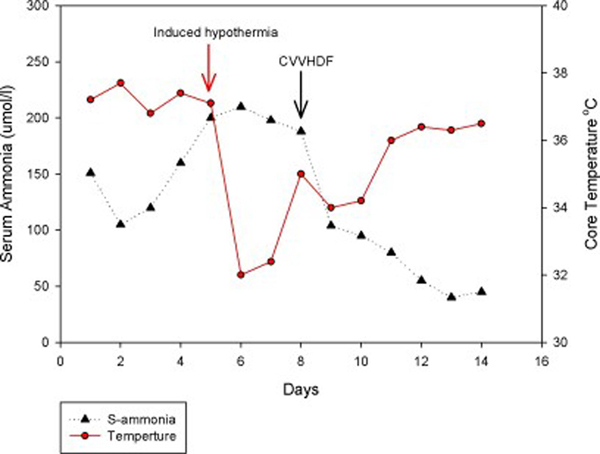
**Time course of arterial ammonia concentration and core body temperature**.

The patient was electively intubated for airway protection and to facilitate endoscopy. He was resuscitated with crystalloids, 5% albumin, packed cells and fresh frozen plasma and treated with vancomycin and piperacillin/tazobactam for presumed sepsis. Ultrasound and Doppler of his upper quadrants was consistent with cirrhosis with normal blood flow and splenomegaly. An upper endoscopy revealed grade 4 esophageal varices with no active bleed. Over the next few days, the patient became progressively unresponsive (off sedation) with his ammonia level rising above 200μmol/L despite aggressive treatment with lactulose and rifaximin. Neurologic assessment revealed posturing to painful stimuli with a poorly reactive pupillary reflex. Computed tomography of the head revealed diffuse white matter edema prominent in the posterior temporal, parietal and occipital lobes. Brain MRI confirmed diffuse white matter edema with temporal and occipital lobe predominance consistent with the diagnostic pattern for PRES (Figure 1 and 2). His course was complicated by the development of tonic-clonic seizures which were controlled with intravenous levetiracetam. His pupils became fixed and non-responsive. Transcranial Dopplers (TCD) of the middle and posterior cerebral arteries demonstrated a marked reduction in cerebral blood velocity consistent with severely increased intracerebral pressure (ICP). As an extraordinary salvage method to control the patient's severe ICP, we lowered his core body temperature to 32°C with the addition of propofol and mannitol, titrated to keep serum osmolarity < 310mmol/L. Induced hypothermia was maintained for 48 hours during which time he regained normal pupillary reflexes with marked improvement in TCD velocities. During the passive rewarming phase, the patient developed massive hematemesis. He required massive transfusion and Minnesota tube placement as attempted banding via endoscopy was unsuccessful. The patient underwent an emergency transjugular intrahepatic portocaval shunt (TIPS) placement followed by repeat induced hypothermia (32 to 34°C). Due to the anticipated increase in the serum ammonia level following the massive gastrointestinal hemorrhage, we initiated high-flow continuous venovenous hemodiafiltration (CVVHD) to facilitate ammonia removal. The CVVHD was associated with a fall in the ammonia level (Figure 3). At this time, the patient was again passively rewarmed and the propofol discontinued. His neurological status improved slowly over the following week becoming more alert and responsive and allowing extubation. A repeat MRI of the brain showed interval improvement in extensive white matter signal abnormality most consistent with resolving PRES. He was discharged home with no neurological sequela apart from amnesia for the entire hospital stay. The patient has returned to work part-time and is currently listed for liver transplantation.

## Discussion

We believe that this is the first documented case of PRES associated with cirrhosis as well as the first report of induced hypothermia for the management PRES and the use of CVVHD for control of hyperammonemia in a cirrhotic patient. The pathogenesis of PRES remains incompletely understood but is probably related to the failure of cerebral autoregulation and endothelial damage [4]. The favored pathogenetic theory suggests autoregulatory disturbance with hyperperfusion, resulting in blood-brain barrier breakdown with reversible vasogenic edema without cerebral infarction [2,4]. However, others have suggested that the triggering event leads to cerebral vasoconstriction, reduced brain perfusion, ischemia and subsequent vasogenic edema [4]-[6]. It is not known why the posterior circulation is preferentially affected. A possible explanation is the lower sympathetic innervation of the posterior cerebral arterial circulation than in the internal carotid territory, with a consequent reduced autoregulation of already impaired cerebral areas [2].

The cause of PRES in our patient and the association with hyperammonemia is unclear. In patients with acute liver failure and severe hyperammonemia, there is gradual cerebral vasodilation due to loss of cerebral auto-regulation resulting in increased cerebral blood flow and vasogenic edema [7]-[10]. Using magnetic resonance diffusion tensor imaging, Kale and colleagues demonstrated an increase in interstitial brain water in patients with cirrhosis and hepatic encephalopathy [11]. In this study, brain water content increased with increasing grade of encephalopathy and decreased with treatment of hyperammonemia. Furthermore, emerging evidence indicates that patients with cirrhosis may have astrocyte swelling and low-grade cytotoxic cerebral edema which is associated with the degree of hyperammonemia [12,13]. Our patient presented with hemorrhage and shock complicated by systemic sepsis and required multiple blood transfusions. Bartynski and colleagues have demonstrated that all these factors may be implicated in the etiology of PRES [14].

Our patient's clinical examination, together with the transcranial Doppler and neuroimaging studies suggested that he had severely increased intracranial pressure (ICP) that required aggressive treatment. Due to the patient's coagulopathy, direct ICP monitoring was considered contraindicated. Moderate induced hypothermia has been shown to reduce cerebral edema following cardiac arrest and in patients with acute liver failure [15,16]. The mechanisms by which hypothermia reduces neuronal injury and cerebral edema are unknown, however, retardation of destructive enzymatic reactions, suppression of free-radical reactions, protection of the fluidity of lipoprotein membranes, reduction in the oxygen demand in low flow regions, reduction in intracellular acidosis, and inhibition of the biosynthesis, release, and uptake of excitatory neurotransmitters have been postulated [17]. Furthermore, in patients with liver disease, hypothermia reduces cerebral ammonia levels by decreasing brain ammonia uptake, decreasing ammonia production and improving ammonia clearance [15]. We believe that the use of induced hypothermia was life saving in our patient. As the cause of PRES was unclear in our patient and because hyperammonemia may cause both vasogenic and cytotoxic edema, we elected to pre-emptively dialyze our patient to reduce the ammonia level. Peritoneal dialysis, hemodialysis, continuous veno-venous hemofiltration (CVVH) and CVVHD have been reported to be helpful in the treatment of hyperammonemia associated with urea cycle disorders in neonates, children and adults. It is unclear whether the use of CVVHDF in our patient contributed to the favorable outcome.

## Consent

Written informed consent was obtained from the patient for publication of this case report and any accompanying images. A copy of the written consent is available for review by the Editor-in-Chief of this journal.

## Competing interests

The authors declare that they have no competing interests.

## Authors' contributions

All authors were directly involved in the care of this patient, were responsible for researching the literature and writing the case report and take responsibility for the final version.
